# Supplementary material for: Behavioral and Immunohistochemical Evidence for Suppressive Effects of Goshajinkigan on Salicylate-Induced Tinnitus in Rats
Source: Brain Sci. 2022 Apr 30;12(5):587. doi: 10.3390/brainsci12050587 (PMC9139011; doi:10.3390/brainsci12050587)
Supplement: Supplementary file 1 [file brainsci-12-00587-s001.zip › brainsci-1687664-supplementary.pdf]

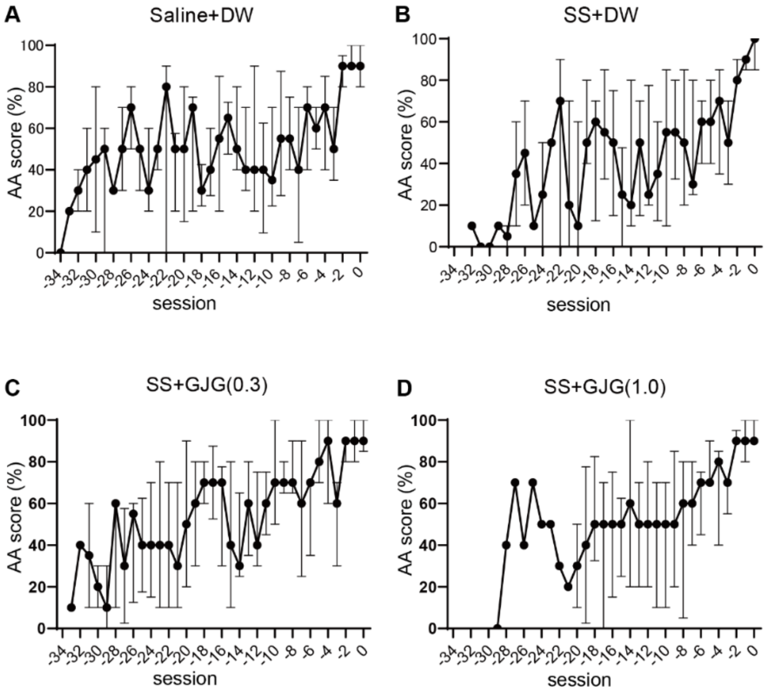

Supplementary Figure S1. Learning curves before behavioral tests. Rats are conditioned until their active avoidance (AA) score is at least 80% for three consecutive sessions. The horizontal axis indicates the number of sessions undergone before conditioning, with session 0 being the third session in which they succeeded three consecutive times. (**A**) Saline + distilled water (DW) group. (**B**) Sodium salicylate (SS) + DW group. (**C**) SS + GJG (0.3) group. (**D**) SS + GJG (1.0) group. Data are presented as median (± interquartile range), n = 9 in each group.
